# Right-Sided Diverticulitis: A Rare Cause of Right-Sided Abdominal Pain

**DOI:** 10.7759/cureus.37123

**Published:** 2023-04-04

**Authors:** Athanasios Papatriantafyllou, Paraskevi Dedopoulou, Konstantina Soukouli, Ioannis Karioris, Stylianos Tsochatzis

**Affiliations:** 1 Department of Surgery, General Hospital of Patras, Patras, GRC

**Keywords:** acute appendicitis, abdominal pain, diverticular disease, right-sided diverticulitis, acute diverticulitis

## Abstract

Acute diverticulitis is a particularly common medical entity, and its frequency increases with age. The most commonly affected part of the large intestine is the sigmoid colon, while right-sided diverticulitis is very rare. Here, we report the case of a 59-year-old man who presented to the emergency department due to acute right lower quadrant abdominal pain. The patient was diagnosed with a computed tomography scan of the abdomen with intravenous contrast with right-sided diverticulitis. The patient’s treatment included hydration and intravenous antibiotics (ciprofloxacin and metronidazole). After three days of hospitalization, the patient was discharged from the hospital in stable condition and without signs of inflammation. This case report demonstrates the importance of including right-sided diverticulitis in the differential diagnosis of acute right lower quadrant abdominal pain, as in most cases patients are treated conservatively without the need for surgical intervention.

## Introduction

Diverticular disease is particularly common in older individuals in Western populations and affects approximately 65% of the population by the age of 85 [1]. Most patients remain asymptomatic throughout their lifetime, and the diagnosis is made only as an incidental finding on endoscopy or cross-sectional imaging [2]. Factors such as increasing age, low intake of dietary fiber, reduced physical activity, obesity, smoking, heavy alcohol consumption, and the use of specific medications increase the chance of developing acute diverticulitis [3]. In patients with abdominal pain, acute diverticulitis should be considered a common cause of the pain [4]. Acute diverticulitis can manifest in different ways, ranging from mild abdominal pain to more severe situations such as peritonitis [1]. The prevalence of diverticular disease affecting the left colon is higher in Western countries compared to Asia, where right-sided diverticular disease is common. Of note, the incidence of right-sided diverticulitis in Western countries is low, accounting for only 1.5% of cases [5]. Here, we report a rare case of acute right-sided diverticulitis in a 59-year-old man.

## Case presentation

**Figure 1:**
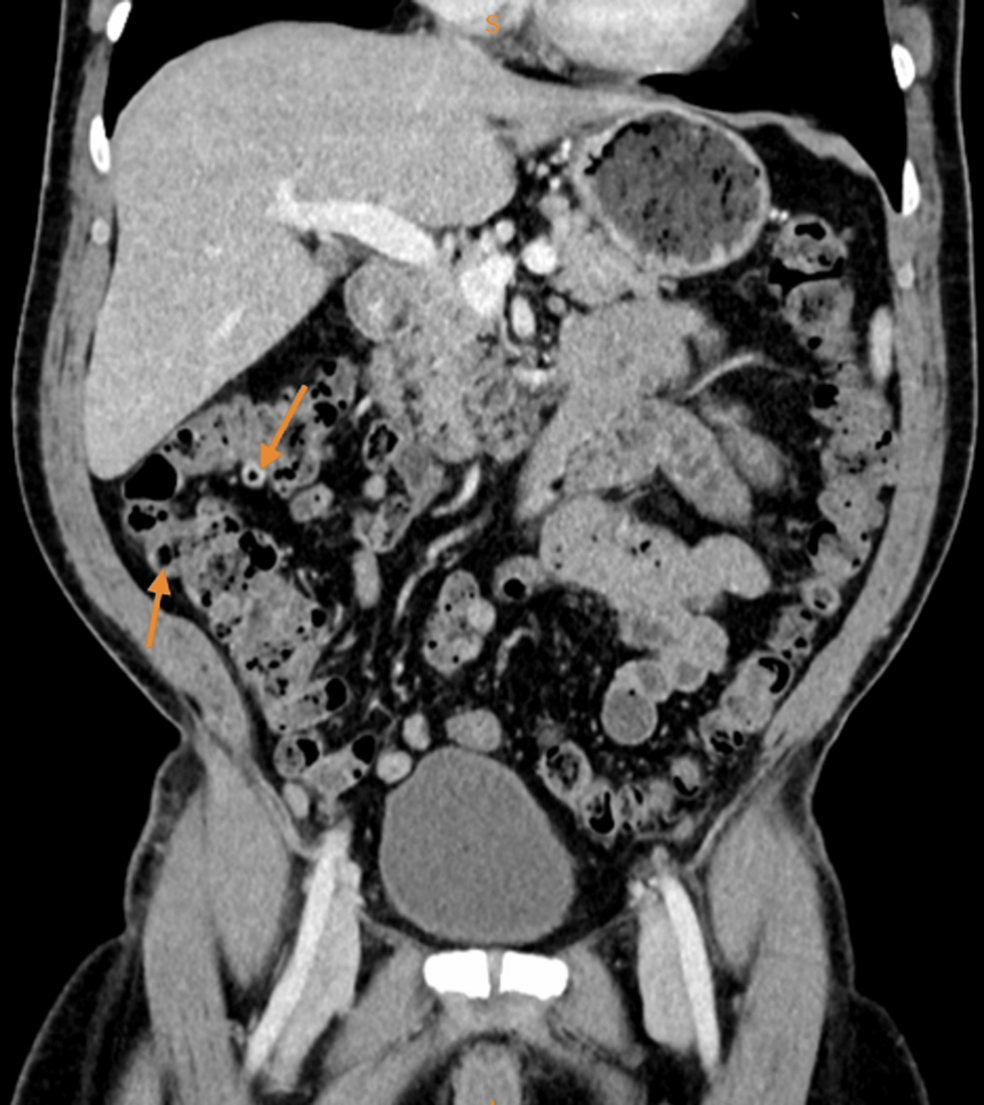
CT coronal scan demonstrating right-sided diverticulitis.

**Figure 2:**
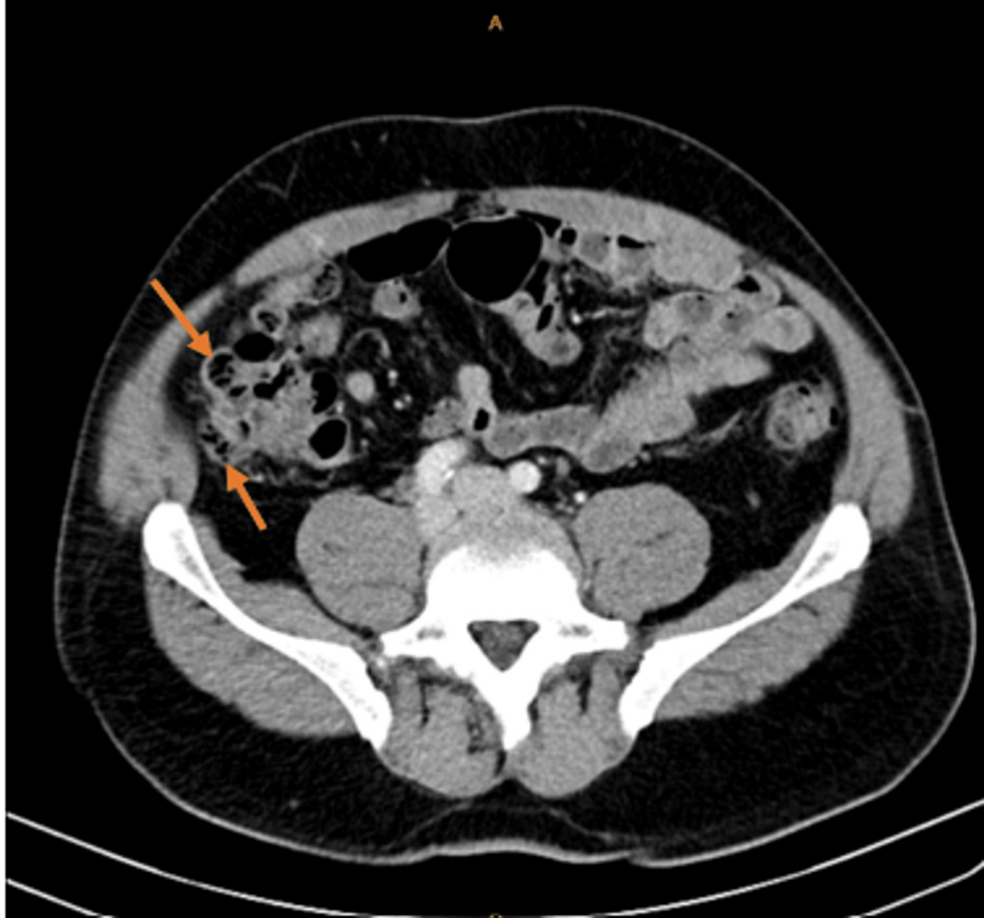
CT axial scan demonstrating right-sided diverticulitis.

A 59-year-old Caucasian man presented to the emergency department with a five-hour history of abdominal pain in the right lower quadrant, which was not associated with nausea or vomiting. He denied any history of fever or urinary symptoms. His past medical history included dyslipidemia, and his past surgical history included a hydrocele repair in early life. In the emergency department, blood pressure was 126/78 mmHg, pulse rate was 87 beats/minute, respiratory rate was 16 breaths/min, oxygen saturation was 98% on room air, and temperature was 36.6°C. Physical examination revealed tenderness in the right lower quadrant, rebound tenderness, and a positive McBurney sign. On laboratory testing, hematocrit was 42.9% (normal: 40.5-47.0%), white cell count was 9.3K/μL (normal: 4.0-10.0K/μL), C-reactive protein was 0.1 mg/dL (normal: 0.0-0.5 mg/dL), urea was 35 mg/dL (normal: 10.0-45.0 mg/dL), and creatinine was 1.0 mg/dL (normal: 0.6-1.3 mg/dL). Diverticula were discovered throughout the colon on computed tomography (CT) scan of the abdomen with intravenous contrast as well as focal thickening throughout the ascending colon to the right colic flexure. Additionally, there was heterogeneity and inflammatory signs in peri-diverticular and pericolic adipose tissue (Figures 1, 2). The appendix showed no evidence of inflammation, wall thickening, or dilatation. These changes were suggestive of non-specific inflammation in the ascending colon and normal appendix.

The patient was admitted to the surgery floor after a comprehensive clinical evaluation, which included a full history, physical examination, and review of laboratory and imaging results. Intravenous ciprofloxacin 400 mg twice a day, intravenous metronidazole 500 mg every eight hours, and intravenous hydration were started at the time of the admission. He was kept for three days without any oral intake. The patient was discharged three days after admission in stable condition without signs of inflammation and was able to tolerate an oral diet. The medical instructions included continuing the oral antibiotic medication and undergoing a colonoscopy in two months.

## Discussion

While left-sided diverticulitis is a common entity in the Western population, right-sided diverticulitis is rare [6]. A high frequency of right-sided diverticulitis has been observed in Asian populations. Genetic factors, Western lifestyle, and diet are assumed to have an impact on the onset of the disease [1].

It is very important to adequately assess every patient with right lower quadrant abdominal pain and tenderness. Differential diagnoses include acute appendicitis, nephrolithiasis, pyelonephritis, inflammatory bowel disease, inguinal hernia, ectopic pregnancy, and a ruptured ovarian cyst. Differentiation of diverticulitis from acute appendicitis is difficult based solely on physical examination and laboratory parameters. Ultrasound and CT are useful tools that help distinguish between the two clinical entities [7].

Acute diverticulitis can be further classified as uncomplicated and complicated. Pelvic abscess, intestinal perforation, bowel fistula, bowel obstruction, peritonitis, or sepsis are potential complications of acute diverticulitis [4]. Surgical intervention may be necessary for patients suffering from complicated acute diverticulitis. Consequently, appropriate handling of acute diverticulitis is essential.

Right-sided diverticulitis and left-sided diverticulitis are considered distinct diseases, and right-sided diverticulitis is less severe than left-sided diverticulitis. It has been observed that patients with right-sided diverticulitis are more likely to be younger and male. The aforementioned patients tend to have less severe disease, while the recurrence rate is lower. On the contrary, patients with left-sided diverticulitis present with more severe disease and worse laboratory findings, such as higher levels of C-reactive protein and creatinine, as well as lower levels of hemoglobin and albumin. However, separate guidelines for right-sided diverticulitis do not exist [8]. Conservative therapy is indicated for uncomplicated acute diverticulitis. The use of antibiotic medication for the treatment of uncomplicated acute diverticulitis is crucial [1]. The issue of administering antibiotic medication is controversial and selective administration is recommended among patients with uncomplicated acute diverticulitis [4].

## Conclusions

Right-sided diverticulitis is a rare clinical entity, and its presentation with abdominal pain in the right lower quadrant, tenderness, nausea, or vomiting can lead to a misdiagnosis of acute appendicitis. In the case of uncomplicated right-sided diverticulitis, conservative therapy is indicated, and the patient avoids an unnecessary surgical intervention. Consequently, clinicians should include right-sided diverticulitis in the differential diagnosis.
